# Environmental Effects on the Electrical Characteristics of Back-Gated WSe_2_ Field-Effect Transistors

**DOI:** 10.3390/nano8110901

**Published:** 2018-11-03

**Authors:** Francesca Urban, Nadia Martucciello, Lisanne Peters, Niall McEvoy, Antonio Di Bartolomeo

**Affiliations:** 1 Physics Department “E. R. Caianiello” and Interdepartmental Centre NanoMates, University of Salerno, via Giovanni Paolo II n. 132, 84084 Fisciano, Italy; furban@unisa.it; 2 CNR-SPIN Salerno, via Giovanni Paolo II n. 132, 84084 Fisciano, Italy; nadia.martucciello@spin.cnr.it; 3 AMBER & School of Chemistry, Trinity College Dublin, 2 Dublin, Ireland; petersli@tcd.ie (L.P.); nmcevoy@tcd.ie (N.M.E.)

**Keywords:** 2D materials, field effect transistors, PMMA, tungsten diselenide

## Abstract

We study the effect of polymer coating, pressure, temperature, and light on the electrical characteristics of monolayer ${\mathrm{WSe}}_{2}$ back-gated transistors with Ni/Au contacts. Our investigation shows that the removal of a layer of poly(methyl methacrylate) (PMMA) or a decrease of the pressure change the device conductivity from p- to n-type. From the temperature behavior of the transistor transfer characteristics, a gate-tunable Schottky barrier at the contacts is demonstrated and a barrier height of ~70 meV in the flat-band condition is measured. We also report and discuss a temperature-driven change in the mobility and the subthreshold swing that is used to estimate the trap density at the ${\mathrm{WSe}}_{2}{/\mathrm{SiO}}_{2}$ interface. Finally, from studying the spectral photoresponse of the ${\mathrm{WSe}}_{2}$, it is proven that the device can be used as a photodetector with a responsivity of $\sim 0.5{\;\mathrm{AW}}^{-1}$ at 700 nm and $0.37\;\mathrm{mW}/{\mathrm{cm}}^{2}$ optical power.

## 1. Introduction

The continuous downscaling of the channel length and thickness in modern field effect transistors (FETs) has increased the need for atomically-layered materials to minimize short channel effects at extreme scaling limits [1,2]. Layered transition metal dichalcogenides (TMDs), owing to their two-dimensional structure, reasonable charge-carrier mobilities, and the absence of dangling bonds can enable extreme channel length scaling and have recently emerged as promising materials for future electronic and optoelectronic devices [3,4,5,6,7]. These graphene-like materials offer the advantages of sizeable and non-zero bandgap, high on/off ratio and quasi-ideal subthreshold swing, mechanical flexibility, and thermal and chemical stability. Similar to graphene, their electronic transport properties are strongly influenced by the choice of the metal contacts [8,9,10], by interface traps and impurities [11,12], as well as by structural defects and environmental exposure [13,14,15,16,17]. These effects need to be understood and controlled for technological applications. 

Molybdenum disulfide (${\mathrm{MoS}}_{2}$) has been one of the most heavily investigated systems from the TMD family to date [18,19,20,21,22,23,24,25]. Similar to ${\mathrm{MoS}}_{2}$, tungsten diselenide (${\mathrm{WSe}}_{2})$, whose electrical and optical properties have been relatively less explored [26], is characterized by an indirect bandgap (1.0–1.2 eV) in the bulk form and shows a transition to a direct gap of 1.6 eV when it is thinned to monolayer [27]. Recent reports on ${\mathrm{WSe}}_{2}$ FETs have demonstrated a relatively high field-effect mobility controllable by temperature and bias voltage [26,28], an ideal subthreshold swing ~60 mV/dec [29] and an *on/off* ratio up to $10^{8}$. The ambipolar behavior, controllable using different metal contacts, like In or Pd [8], which favor electron and hole injection, respectively [26,30,31], makes mono- and few-layer ${\mathrm{WSe}}_{2}$ an interesting material for complementary logic applications; indeed, a stable ${\mathrm{WSe}}_{2}$-based CMOS technology has been demonstrated [32,33,34].

A great challenge for electronic integration of ${\mathrm{WSe}}_{2}$ is the development of low-resistance ohmic contacts, a task often complicated by the appearance of Schottky barriers due to the occurrence of Fermi level pinning [35,36]. Accordingly, several studies have aimed to clarify the role of the contacts, focusing on the carrier transport at the ${\mathrm{WSe}}_{2}$/metal interface [30,37,38]. 

In this paper, we study back-gated monolayer${\;\mathrm{WSe}}_{2}$ devices with Ni contacts, measuring their electrical characteristics under different conditions, considering, for instance, the effect of a poly(methyl methacrylate) (PMMA) coating layer, and the dependence on the chamber pressure and the sample temperature. Similar to graphene [39,40], we observe that PMMA strongly influences the electrical transport, in this case to the extent that the polarity of the device changes from p-type to n-type conduction when the PMMA layer is removed. We demonstrate that lowering the pressure on air-exposed ${\mathrm{WSe}}_{2}$ FETs affects their characteristics in a similar way to PMMA, turning the conduction from p- to n-type. Furthermore, from the current-voltage (I-V) characteristics measured at different temperatures, we prove a gate modulation of the Schottky barrier (SB) at the contacts.

In addition, we study the temperature dependence of the carrier mobility and the subthreshold swing and show that both undergo a change of behavior with increasing temperature. From the subthreshold swing data, we derive the interface trap density, which affects the photoresponse of the device. The monolayer $WSe_{2}$ device, characterized at several laser wavelengths, achieves a responsivity as high as $\sim 0.5\;AW^{-1}$ at 700 nm, i.e. at a photon energy close to the ${\mathrm{WSe}}_{2}$ bandgap.

## 2. Experimental

The ${\mathrm{WSe}}_{2}$ flakes were grown in a two-zone heating furnace. Selenium pellets (Sigma-Aldrich Inc, St. Louis, MO, USA) were evaporated at 250 °C in the lower-temperature, upstream heating zone, while in the high-temperature, downstream zone the tungsten precursor (20 nm sputtered and subsequently oxidized tungsten) was placed with the growth substrate. A highly p-doped Si (silicon) substrate covered by 300 nm of ${\mathrm{SiO}}_{2}\;$(silicon dioxide) was placed top down on the tungsten precursor, which was heated up to 850 °C. The tungsten precursor/growth substrate stack forms a microreactor, which increases the reactivity due to the close proximity between the precursor and growth substrate, requiring a lower amount of precursor and minimizing the contamination of the furnace. This is a similar approach to previous reports on the growth of ${\mathrm{MoS}}_{2}$ in a microreactor [41] but in this case different chalcogen and transition metal precursors are used. Both furnaces were kept at the reaction temperatures for 40 min under a flow of 50 sccm forming gas ($H_{2}$/Ar 1:9) at a pressure of 6 Torr, after which the furnace was cooled down.

**Figure 1:**
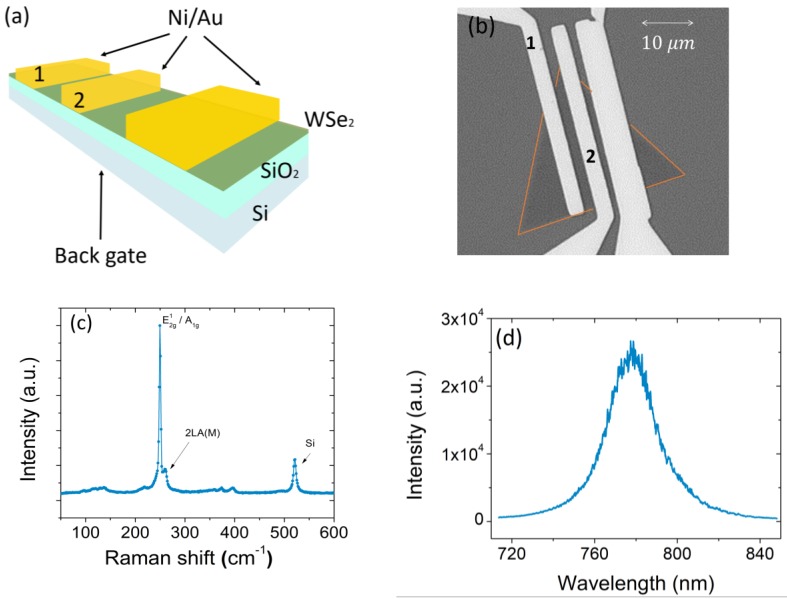
Schematic diagram (**a**) and optical microscope image (**b**) of the $WSe_{2}$ back gate FET transistor. Raman (**c**) and photoluminescence (**d**) spectra of the ${\mathrm{WSe}}_{2}$ flake.

A schematic of the back-gated FET device and a scanning electron microscope top-view of a${\;\mathrm{WSe}}_{2}$ monolayer with evaporated Ni/Au (5/50 nm) contacts, made by use of e-beam lithography, are shown in Figure 1a,b. In the following, the transistor characterization refers to contact 1 and 2, which define a device with channel length L~2 μm and mean width W~22 μm (Figure 1b). The electrical analyses are performed using a Keithley 4200 SCS (semiconductor characterization system, Tektronix Inc., Beaverton, OR, USA) connected with a Janis ST-500 probe station (Janis Research Company LLC, Woburn, MA, USA), equipped with four probes used for the electrical connection to the drain and source Ni/Au terminals and to the Si back-gate of the device. 

Raman and photoluminescence (PL) spectra were acquired using a WITec Alpha 300 tool (WITec GmbH, Ulm, Germany) with a 532 nm excitation laser. The Raman spectrum of the ${\mathrm{WSe}}_{2}$, displayed in Figure 1c, exhibits two peaks around $\sim 250{\;\mathrm{cm}}^{-1}$ and $\sim 260{\;\mathrm{cm}}^{-1}$, corresponding to an overlapping contribution from the in-plane vibrations of W and Se atoms (E^1^_2g_) and out-of-plane vibrations of Se atoms (A_1g_), and to a second-order resonant Raman mode (2 LA (M)) due to LA phonons at the M point in the Brillouin zone [42,43], respectively. The peak frequency positions are typical of a ${\mathrm{WSe}}_{2}$ monolayer of thickness d~0.7 nm  [29]. The monolayer structure of the flake is further confirmed by the PL spectrum of Figure 1d, which shows an intense and narrow peak with maximum at ~778 nm and FWHM of ~21 nm. Such a peak corresponds to a bandgap of ~1.59 eV, a value closer to that of a monolayer than to that of a bilayer. Hence, both Raman and PL spectra indicate that the flake is a monolayer.

## 3. Results and Discussion

We start the transistor characterization by comparing the device I-V curves with and without a PMMA coating layer, which was used to protect the transistor channel from residue and adsorbates [44,45]. It has been observed that a PMMA film, or even only residue of it, can cause p-type doping of graphene and other 2D channels due to the presence of oxygen. Here, we report a similar effect for CVD-grown ${\mathrm{WSe}}_{2}$ FETs, measured at T=293 K, and P~2 mbar. 

**Figure 2:**
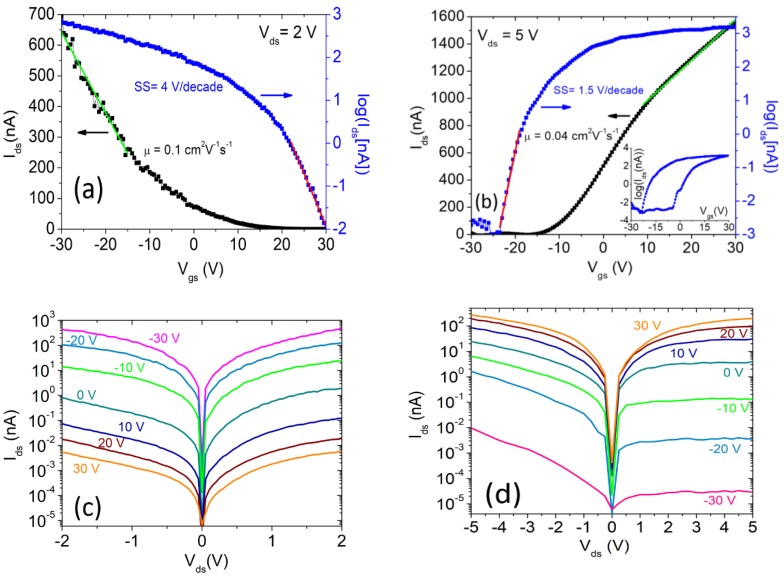
Transfer characteristics ($I_{ds}-V_{gs}$ curves) obtained at a drain voltage bias $V_{ds}=2\;V$ for the device covered with PMMA (**a**) and after the removal of PMMA (**b**) at $V_{ds}=5\;V$. The inset shows a complete cycle with the gate voltage $V_{gs}$ swept forward and backward. Output characteristics ($I_{ds}-V_{ds}$ curves) at different gate voltages for the device with (**c**) and without (**d**) PMMA. For the uncovered device, the drain bias was increased from $V_{ds}=2\;V$ to $V_{ds}=5\;V$ to better characterize the above-threshold region.

The PMMA-covered devices behave like p-type transistors, as can be seen from the $I_{ds}-V_{gs}$ transfer curves of Figure 2a which show high channel current $I_{ds}$ (*on*-state of the FET) at negative gate voltages, $V_{gs}$. The p-type conduction is explained considering the charge transfer to oxygen which, acting as electron capture center, suppresses the free electron density and enhances the hole concentration in the channel. Furthermore, the transition of the channel to p-type could cause a depinning of the Fermi level and facilitate hole injection at the contacts (indeed, in TMDs, Fermi level pinning often occurs close to the minimum of the conduction band) [36,46,47,48]. After the removal of the PMMA by immersion in acetone, a dramatic change to n-type behavior appeared, with the *on*-state at $V_{gs}<0\;V$, as shown in Figure 2b. A similar effect has been reported in literature [32,49] for exfoliated $WSe_{2}$ flakes on an ${\mathrm{SiO}}_{2}/\mathrm{Si}$ substrate covered by $F_{4}\mathrm{PCNQ}$-doped PMMA.

The corresponding $I_{ds}-V_{ds}$ output characteristics are reported in Figure 2c,d. Both plots show non-linear behavior for the device in the *on* state with increasing positive-negative asymmetry when the device approaches the *off* state. This points to the presence of Schottky barriers at the ${\mathrm{Ni}/\mathrm{WSe}}_{2}$ contacts, possibly with slightly different heights [37,50]. 

For increasing $V_{gs}$, the channel current at constant $V_{ds}$ shows an exponential dependence (below the threshold region) followed by a linear or power law behavior (above the threshold region). 

A quadratic behavior is particularly evident in the transfer characteristic of Figure 2a, even though the transistor is operated in the triode region. The parabolic dependence of $I_{ds}$ on $V_{gs}$ can be ascribed to the linear gate-voltage dependence of the mobility μ [51,52], which defines the drain current as:

(1) $$\;I_{ds}=\frac{WC_{ox}\;\mu }{L}\left(V_{gs}-V_{th}\right)V_{ds}\;$$

with:

(2) $$\;\mu =\mu_{B}\left(V_{gs}-V_{th}\right)\;$$

in which $\mu_{B}$ represents the mobility per unit gate voltage and $V_{th}$ is the threshold voltage. The $V_{gs}$-dependent mobility can be explained by considering that the increasing carrier density becomes more effective at screening Coulomb scattering or in filling trap states at higher $V_{gs}$, thus resulting in enhanced mobility. 

The dependence of the mobility on the gate voltage can be established by extracting it in the usual way using:

(3) $$\;\mu =\frac{L}{W}\frac{1}{C_{ox}}\frac{1}{V_{ds}}\frac{dI_{ds}}{dV_{gs}}\;$$

**Figure 3:**
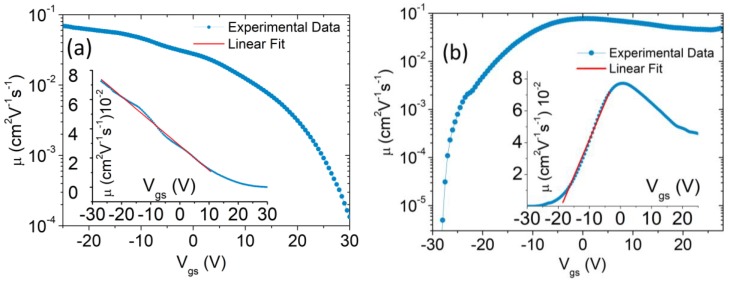
Mobility versus gate voltage on a logarithmic scale for the $WSe_{2}$ flake covered (**a**) and uncovered (**b**) by PMMA. The inset graphs show the mobility on a linear scale.

Figure 3a,b show the $\mu -V_{gs}$ curves on logarithmic and linear (insets) scales obtained from Equation (3) and the data of Figure 2, for the devices with and without PMMA, respectively. These confirm a linear dependence of μ on $V_{gs}$ over a certain range. Remarkably, for the device with removed PMMA, the mobility shows the typical decrease observed in common FETs due to increased scattering suffered by carriers attracted at the channel/dielectric interface at higher gate voltages.

By neglecting the $V_{gs}$ dependence of the mobility, as is usually done in the literature, μ can be obtained by fitting a straight line to the transfer characteristics, as shown in Figure 2a,b. 

By this method, we estimate an electron mobility of $\sim 0.04\;cm^{2}V^{-1}s^{-1}$ for the n-type transistor without PMMA, consistent with other works with ${\mathrm{WSe}}_{2}$ on ${\mathrm{SiO}}_{2}$ [41], and a hole mobility of $\sim 0.1{\;\mathrm{cm}}^{2}V^{-1}s^{-1}$ for the PMMA-covered p-type transistor. We notice that, although a different channel carrier concentration might contribute to this difference, these values are consistent with the higher hole mobility in ${\mathrm{WSe}}_{2}$ reported elsewhere [29,32,45]. The low mobility indicates a high density of trap states, which are also responsible for the hysteretic behavior of the transfer characteristic shown in the inset of Figure 2b. The hysteresis is caused by trapping and detrapping of charge carriers, whose potential adds to that of the back-gate [51,52,53,54]. 

The subthreshold swing, $SS=dV_{gs}/dlog\left(I_{ds}\right)$, is 4 V/decade and 1.5 V/decade, for the p-type and n-type transistor, respectively. The different SS results from a different trap density at the ${\mathrm{WSe}}_{2}/\mathrm{dielectric}$ interface, implying a higher trap density when the ${\mathrm{WSe}}_{2}$ channel is covered by PMMA, which acts as a second interface [55,56]. 

**Figure 4:**
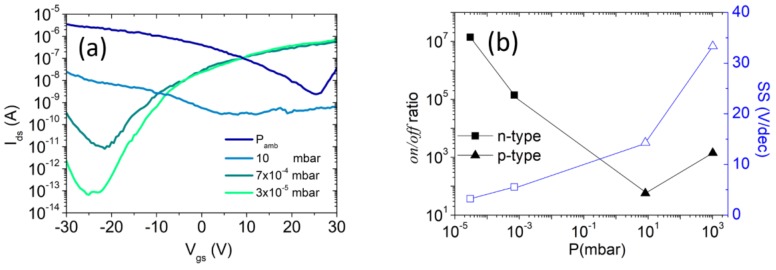
(**a**) Transfer characteristics at different pressures from atmospheric value (blue curve) to $\sim 10^{-5}$ mbar (light green curve) (**b**) *On/Off* ratio (full marks, left scale) and subthreshold swing (empty marks, right scale) as a function of the chamber pressure.

After the removal of the polymeric film and exposure of the device to air for a few days, we observed a restoration of a prevailing p-type behavior due to $O_{2}\;$and water adsorption on the ${\mathrm{WSe}}_{2}$ surface and possible depinning of the Fermi level. We then studied the effect of dynamic pressure by increasing the vacuum level of the probe station chamber from atmosphere (~1 bar) to $\sim 10^{-5}\;\mathrm{mbar}$. As reported in Figure 4a, the transistor transfer characteristic changed again from p- to n-type with a gradual decrease of the subthreshold swing and an increase of the *on/off* ratio, as shown in Figure 4b. We attribute the polarity change to the desorption of adsorbed${\;O}_{2}{\;\mathrm{and}\;H}_{2}O$ and to the consequent possible pinning of the Fermi level close to the minimum of the conduction band.

We then examined the temperature (T) dependence of the transfer characteristics of the PMMA-free, n-type transistor at low pressure, which can be conveniently used to investigate the Schottky barrier for electrons at the contacts.

**Figure 5:**
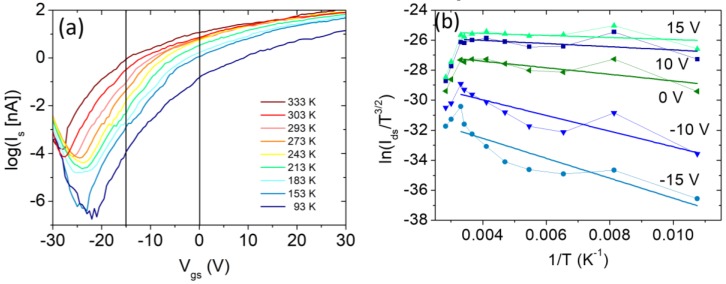

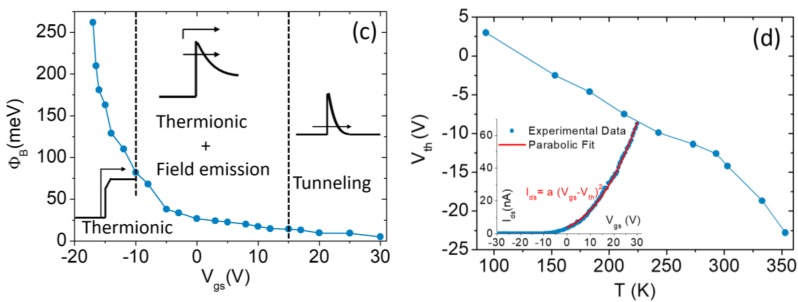
(**a**) Transfer characteristic at several temperatures. (**b**) Arrhenius plot of the current at different temperatures corresponding to a subset of the gate voltages (two of these $V_{gs}\;$values are represented by the vertical lines in (**a**)). (**c**) Apparent Schottky barrier as a function of the gate voltage; the insets show the band alignment and the transport regimes at the Ni/WSe_2_ reverse-biased contact. (**d**) Threshold voltage $V_{th}$ as a function of the temperature; the inset shows, as an example, the parabolic fit of the $I_{ds}-V_{gs}$ curve at T=273 K.

We extract the Schottky barrier at the flat-band condition from a plot of the Schottky barrier height as a function of $V_{gs}\;$for the device at a source-drain bias of 5 V (Figure 5). Given that the device is n-type, such a barrier refers to electron injection from the contacts and it is caused by the aforementioned pinning of the Fermi level close to the minimum of the conduction band. Measuring the $I_{ds}-V_{gs}$ characteristics of the device at several temperatures (Figure 5a) and extracting $I_{ds}-T$ datasets at given gate voltages (examples are marked by the vertical lines in Figure 5a), we constructed the Arrhenius plot of Figure 5b, showing the $ln\left(I_{ds}/T^{3/2}\right)-\frac{1}{T}$ curves at a representative subset of $V_{gs}$ values. We assumed that the contacts behave as two back-to-back Schottky junctions, where the current is controlled by the reverse-biased junction and is written as:

(4) $$\;I_{ds}\sim T^{3/2}\;exp\left(-\frac{\Phi_{B}}{kT}\right)\;$$

where k is the Boltzmann constant, T is the absolute temperature, and $\Phi_{B}$ is the Schottky barrier height [30,57,58]. According to equation (4), a linear fit of $\ln\left(I_{ds}/T^{3/2}\right)$ vs 1/T for each $V_{gs}\;$dataset in Figure 5b yields a Schottky barrier $\Phi_{B}.\;$The so-obtained $\Phi_{B}-V_{gs}$ relationship is displayed in Figure 5c and can be divided into three zones, each one corresponding to a different transport regime, consistent with the behavior of the transfer characteristics of Figure 5a.

At low gate voltage the device is set in the *off* state and the transport is due to the thermal excitation of electrons over the barrier. The ${\mathrm{WSe}}_{2}$ conduction-band level is gradually lowered by the increasing gate voltage, as sketched in the insets of Figure 5c. This results in a lowering of the barrier with a subsequent steep exponential rise of the current in the transfer characteristic (with 60 mV/decade slope in the ideal case). When the gate voltage is further increased the device reaches the flat band condition ($V_{gs}=V_{FB}$), which sometimes appears in the subthreshold part of the transfer characteristics as a sudden change of slope; for $V_{gs}>V_{FB}$ the device enters the so-called Schottky regime which includes part of the downward bended region of $I_{ds}-V_{ds}\;$curves and is characterized by thermionic emission and field emission. Finally, at higher $V_{gs}$, tunneling through the thinned $\mathrm{Ni}/{\mathrm{WSe}}_{2}$ barrier becomes the dominant conduction mechanism and the device reaches the above threshold region with a linear, or power-law, $I_{ds}-V_{gs}\;$dependence.

The gate voltage that corresponds to $V_{FB}$ is identified by the change of slope in the $\Phi_{B}-V_{gs}$ plot at lower $V_{gs}$. The $\Phi_{B}$ corresponding to $V_{gs}=V_{FB}$ is the so-called Schottky barrier height at the flat-band (or simply Schottky barrier). From the plot in Figure 5c, its value is ~70 meV, confirming the presence of a barrier at the Ni-${\mathrm{WSe}}_{2}\;$contacts, inferred from the asymmetric output characteristics of Figure 2. 

Figure 5d shows the temperature-dependent behavior of the threshold voltage $V_{th}$, which has been extracted assuming a quadratic $I_{ds}-V_{gs}$ law as expressed by Equation (1) and (2). The decrease in $V_{th}$ is easily explained by considering that the increasing temperature accelerates the transition from the Schottky to the power-law (above threshold) regime; furthermore, the plot seems to indicate a change of slope above room temperature.

**Figure 6:**
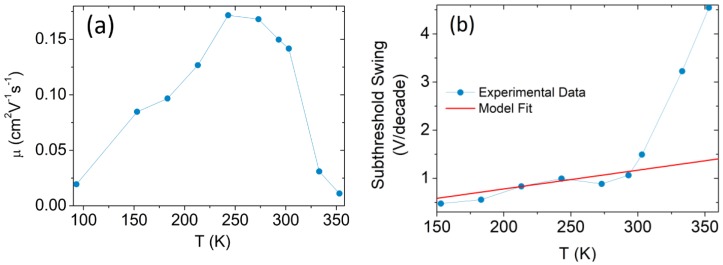
Temperature dependence of (**a**) mobility per unit voltage $\mu_{B}$ and (**b**) subthreshold swing.

Figure 6a reports the temperature-dependent behavior of the mobility, μ, at $V_{gs}=10\;V$ obtained from the quadratic fit of the $I_{ds}-V_{gs}$ curves.

The mobility increases for T<250 K and decreases for T>250 K, behavior typical of semiconductor materials, indicating that charged-impurity Coulomb scattering dominates at lower temperatures, while phonon scattering becomes the conduction-limiting mechanism at high temperature [44]. 

The subthreshold swing has a dependence on temperature that can be simplified with the following expression:

(5) $$\;SS=n\frac{kT}{q}ln10\;$$

where n is the body factor which is related to the interface trap ($C_{it}$), $SiO_{2}\;\left(C_{SiO2}\right)$ and channel depletion layer ($C_{dl}$) capacitances by:

(6) $$\;n=1+\frac{C_{it}+C_{dl}}{C_{ox}}\;$$

Figure 6b confirms the linear SS−T dependence (Equation 5) but shows an unexpected rise above room temperature. The deviation from Equation (5) behavior at high temperature is a consequence of the low Schottky barrier which becomes less effective above room temperature (kT= 26 meV), resulting in an increase of the subthreshold current leakage.

Assuming that the ${\mathrm{WSe}}_{2}\;$monolayer channel is fully depleted, i.e. that $C_{dl}\approx 0$, from the fit of the experimental data with Equation (5), we obtain a n≈48 and an interface trap density $N_{it}=\frac{C_{it}}{q^{2}}\approx 1.3\times 10^{13}\;eV^{-1}cm^{-2}$, which is consistent with previous results reported in the literature [59].

The presence of such a density of trap states explains the observed hysteretic behavior of the transfer characteristic, displayed in the inset of Figure 2b [52]. It also affects the electrical response of the device under illumination. 

**Figure 7:**
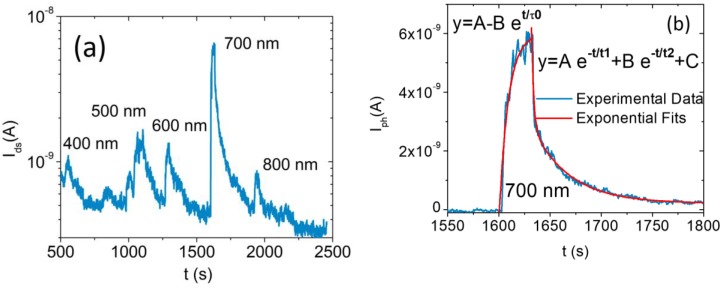
(**a**) Drain-source current measured under 30 s laser pulses at different wavelengths ($V_{ds}=+\;5\;V,$$V_{gs}=0\;V$, $P\sim 10^{-4}\;mbar$). (**b**) Photocurrent generated by a 30 s laser pulse at the wavelength of ~700 nm and optical power $\sim 0.37\;mW/cm^{2}$ with exponential fits.

We performed photocurrent measurements with light at different wavelengths, selected by filtering a supercontinuous laser source (NKT Photonics, Superk Compact, wavelength ranging from 450 nm to 2400 nm, total output power of 110 mW) using pass-band filters with 50 nm bandwidth. Figure 7a shows the photoresponse of the ${\mathrm{WSe}}_{2}$ FET to laser light pulses of 30 s for five different wavelengths. 

The photocurrent exhibits a higher peak at the wavelength of 700 nm (photon energy 1.7 eV), which is slightly above the bandgap of a ${\mathrm{WSe}}_{2}$ monolayer, supporting the Raman and the PL spectroscopy assignment of the single-layer nature of the ${\mathrm{WSe}}_{2}$ channel.

Figure 7b reports the photocurrent, $I_{ph}=I_{light}-I_{dark}$, obtained in response to a laser pulse of 30 s at a wavelength of ~700 nm, and an optical power $\sim 0.37\;mW/cm^{2}$. It corresponds to a peak with rising time $\tau_{0}\sim 9\;s$ and a double exponential decay with times $\tau_{1}\sim 2\;s$ and $\tau_{2}\sim 36\;s$, indicating the presence of faster and slower traps [60]. Such features are consistent with a photoresponse decay longer than 5 s for quasi-ohmic contacts measured on similar ${\mathrm{WSe}}_{2}$ FETs [61,62]. Indeed, we notice that the contact type can play an important role in the response time of ${\mathrm{WSe}}_{2}$ phototransistors and that reduced times have been reported for Schottky contacts [61,62]. 

Furthermore, we estimate a photoresponsivity (Figure 7b):

(7) $$\;R=\frac{I_{ph}}{W_{opt}}\approx 0.5\frac{A}{W}\;$$

where $W_{opt}$ is the incident power. This is in good agreement with the previously reported value of 0.6 A/W obtained at 750 nm [62]. Such a responsivity is competitive with solid state devices on the market and, despite the ultrathin nature of the absorber, confirms the excellent photoresponse of monolayer ${\mathrm{WSe}}_{2}\;$due to its direct bandgap [63,64].

## 4. Conclusions

We showed that different environmental conditions can have dramatic effects on the electrical properties of back-gated transistors with monolayer ${\mathrm{WSe}}_{2}\;$channels. In particular, we demonstrated that the removal of a polymer coating layer, as well as of oxygen and water adsorbates, can change the conduction from p- to n-type. From I-V characterization at different temperatures, we extracted the $\mathrm{Ni}/{\mathrm{WSe}}_{2}$ Schottky barrier height, which we studied as a function of the back-gate voltage. We reported and discussed a change in the temperature behavior of the mobility and the subthreshold swing. Finally, we studied the photoresponse of the device to selected laser wavelengths achieving a responsivity competitive with solid-state devices on the market.
